# Early life determinants induce sustainable changes in the gut microbiome of six-year-old children

**DOI:** 10.1038/s41598-019-49160-7

**Published:** 2019-09-03

**Authors:** Silvia Gschwendtner, Hyena Kang, Elisabeth Thiering, Susanne Kublik, Bärbel Fösel, Holger Schulz, Susanne Krauss-Etschmann, Joachim Heinrich, Anne Schöler, Michael Schloter, Marie Standl

**Affiliations:** 1 Research Unit for Comparative Microbiome Analysis, Helmholtz Zentrum München, German Research Center for Environmental Health, Ingolstädter Landstr. 1, 85764 Neuherberg, Germany; 2 Institute of Epidemiology, Helmholtz Zentrum München, German Research Center for Environmental Health, Ingolstädter Landstr. 1, 85764 Neuherberg, Germany; 3 Division of Metabolic and Nutritional Medicine, Dr. von Hauner Children’s Hospital, University of Munich Medical Center, Lindwurmstraße 4, 80337 Munich, Germany; 4 Institute for Experimental Medicine, Christian-Albrechts-Universität zu Kiel, Niemannsweg 11, 24105 Kiel, Germany; 5 Division of Experimental Asthma Research, Research Center Borstel, Leibniz Lung Center, Member of the German Center for Lung Research (DZL), Parkallee 1-40, 23845 Borstel, Germany; 6 Institute and Clinic for Occupational, Social and Environmental Medicine, University Hospital, LMU Munich, Ziemssenstr. 1, 80336 Munich, Germany; 7 Institute for Neuropathology, Charité, Charitéplatz 1, 10117 Berlin, Germany; 8 German Cancer Consortium (DKTK), Heidelberg, Germany; 9 German Cancer Research Center (DKFZ), Im Neuenheimer Feld 280, 69120 Heidelberg, Germany; 10 Technical University of Munich, Zentral Insititut for Food and Health (ZIEL), Weihenstephaner Berg 1, 85354 Freising, Germany

**Keywords:** Applied microbiology, Risk factors

## Abstract

While the association between early life determinants and the development of the gut microbiome composition in infancy has been widely investigated, a potential persistent influence of early life determinants on the gut microbial community after its stabilization at later childhood remains largely unknown. Therefore, we aimed to identify the association between several early life determinants and the gut microbiome composition in six-year-old children from the LISA birth cohort. A total number of 166 fecal samples were analyzed using 16S rRNA gene-based barcoding to assess bacterial diversity pattern. The bacterial profiles were investigated for their association with maternal smoking during pregnancy, mode of delivery, breastfeeding, antibiotic treatment between one and two years of age, gender and socioeconomic status (SES). While alpha and beta diversity of the infants’ gut microbiome remained unaffected, amplicon sequence variants (ASVs) annotated to Firmicutes and Actinobacteria responded to early life determinants, mostly to feeding practice and antibiotics use. ASVs associated to Bacteriodetes remained unaffected. Our findings indicate that early life determinants could have a long-term sustainable effect on the gut microflora of six-year-old children, however, associations with early life determinates are weaker than reported for infants.

## Introduction

The human gut microbiome triggers many functions that are essential for our health and wellbeing, including education of the immune system, formation of a barrier against pathogens and digestion of indigestible nutrients^1,2^. Not surprisingly, changes in the gut microbiome are associated with the development of diseases related to acute and chronic inflammation, allergy or immune dysregulation^3–5^. As consequence, the gut microbiome could be a potential target of new preventive therapeutic strategies. Thus, understanding the factors that induce a dysbiosis of the gut microbiome is essential to improve human health.

Colonization of the gut starts at birth as result of transfer of microbes from the mother to the infant^6^. Besides transferred maternal gut microbiota, bacteria from the maternal vagina act as inoculum of the gut microbiome of spontaneously delivered newborns, whereas infants delivered by C-section receive their gut microbiome both from the skin of the mother and from the hospital environment due to absence of direct contact with the vagina^7^. After birth, breastfeeding is the first dietary source which is thought to stimulate the development of a highly diverse microbiome^8^, mainly as result of oligosaccharides, which are part of the mother’s milk and promote the growth of beneficial bacteria^9^. During the subsequent years, the gut microbiome gradually develops its structure and function^10^. Here a number of factors are considered as major drivers, including besides genetic disposition also food quality, pollutants and medication^4,11^. Mainly antibiotic therapy may disturb the natural development process and beneficial microbes might be replaced by pathogens and pathobionts^12^.

Due to the importance of the gut microbiome for human health, there is a strong need to develop strategies for a well-controlled development of the gut microbiome during childhood. To achieve this goal a detailed understanding of the long-term impact of early life determinants on the gut microbiome composition is required. However, most studies so far focused only on early infancy^7–9,12^ and data on long-term development of the gut microbiome during childhood are missing. Therefore, we aimed to investigate the role of early life determinants, which have been related to later health (maternal smoking during pregnancy, mode of delivery, breastfeeding, antibiotics treatment in infancy, gender and socioeconomic status (SES)) on the long-term development of the gut microbiome during childhood and focused on six-year-old children of a large birth cohort follow-up which was established to investigate effects of lifestyle factors on the development of immune system and allergies in Germany.

## Results

### Major characteristics of the studied children

The analyzed samples comprised 44% (*n* = 73) females. 71% (*n* = 111) of the children were exclusively breastfed (eBF) for at least four months. 16% (*n* = 27) of the children were delivered by C-section and 51% (*n* = 84) of the children were treated with antibiotics (AB) between the age of one and two years. 11% (*n* = 18) of the mothers smoked during pregnancy. 66% (*n* = 109) of the mothers had a high socioeconomic status (SES). To determine the representativeness of these samples for the LISA study population the analyzed subset and the remaining LISA birth cohort from the Munich study center were compared and did not show any significant differences regarding the early life determinants and potential confounding factors (S2 data). Thus, we assume that the analyzed subjects are representative of the entire cohort.

### Impact of early life determinants on gut microbiome composition

**Figure 1:**
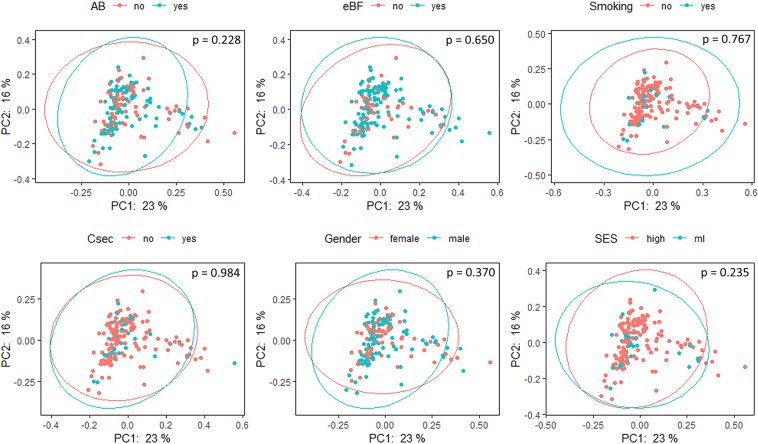
PCoA plot of beta diversity of the gut microbiome based on weighted Unifrac distances for the investigated early life determinants. Ellipses show the two groups of the early life determinants (antibiotics use (AB): yes/no, exclusive breastfeeding (eBF): yes/no, smoking during pregnancy: yes/no, C-section (Csec): yes/no, gender: female/male and socioeconomic status (SES): high/ml) under investigation (n = 166).

A total of 18,599,243 raw reads were obtained. After quality filtering and chimera removal via DADA2 5,185,163 high-quality partial 16S rRNA gene sequences remained which were assigned to 4,888 unique amplicon sequence variants (ASVs). To exclude potential contaminants, all 15 ASVs occurring in extraction and PCR no template controls were removed from the dataset. To compare samples without statistical bias, subsampling with 4918 reads was performed, reflecting the lowest read number obtained per sample. Rarefaction analysis indicated diversity coverage of >99% (S3 data) and consequently an adequate sampling depth for further analysis. Alpha diversity of the gut microbiome for each early life determinant subgroup was determined by calculating richness, evenness, Shannon and Simpson index. Overall, none of the diversity indices was significantly affected by early life determinants (except exclusively breastfeeding which significantly decreased evenness, p = 0.047) (Table 1). Further multivariate analysis of variance based on Yue Clayton dissimilarity, Euclidean distance of Hellinger transformed data, Jaccard distance, Bray-Curtis distance and both weighted and unweighted Unifrac distance showed that none of the early life determinants significantly influenced the beta diversity of the gut microbiome composition (Fig. 1, showing PCoA of weighted Unifrac distance as example).

**Table 1:** Alpha diversity indices of the gut microbiome for the investigated early life determinants tested (antibiotics use during age 1–2 years, exclusively breastfeeding (eBF), smoking during pregnancy, C-section, gender (female/male) and socioeconomic status (SES; high/ml)) (n = 166).

	Antibiotics		eBF		Smoking		C-section		Gender		SES	
	no	yes	no	yes	no	yes	no	yes	F	M	high	ml
Richness	169	160	159	168	164	166	163	171	157	171	165	164
Eveness	0.81	0.82	**0.83**	**0.81**	0.82	0.80	0.81	0.82	0.81	0.81	0.81	0.81
Shannon	4.12	4.13	4.16	4.11	4.13	4.06	4.12	4.16	4.08	4.16	4.13	4.11
Simpson	0.96	0.97	0.97	0.96	0.96	0.96	0.96	0.96	0.96	0.96	0.96	0.96

Significant differences were calculated using multivariate ANOVA and are indicated by bold letters (*p* < 0.05).

### Taxonomic differences associated with early life determinants

**Figure 2:**
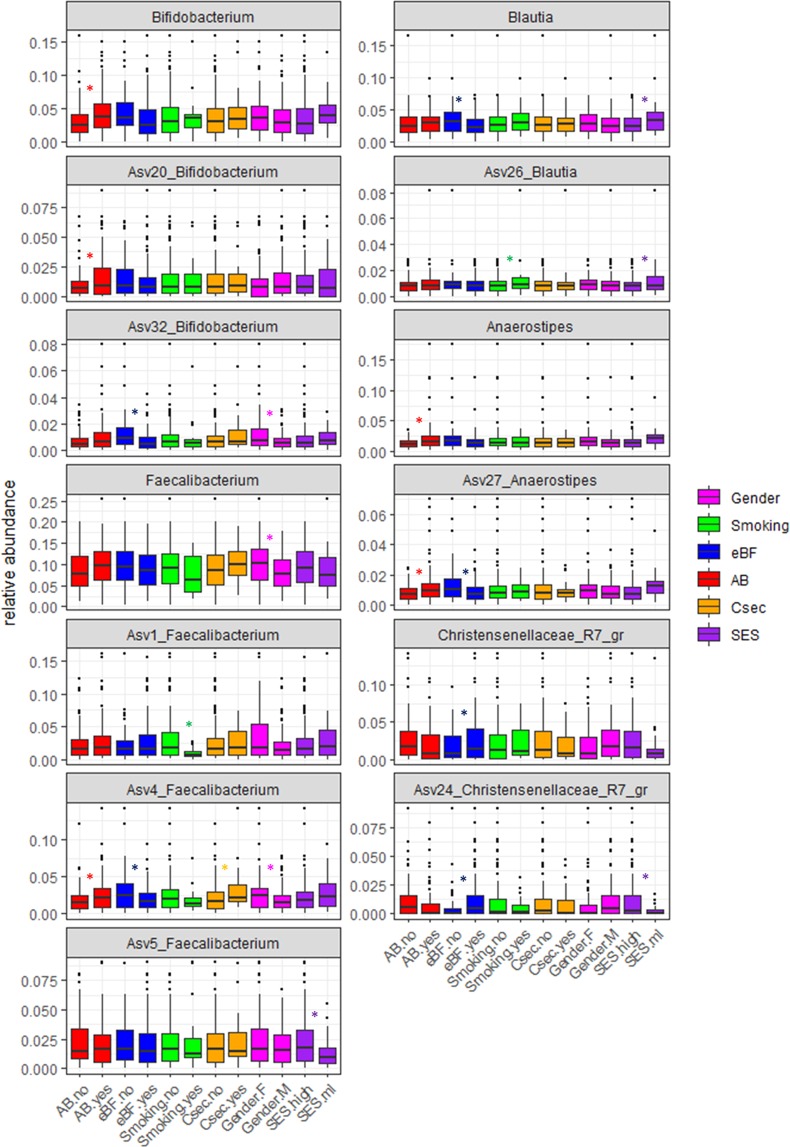
Relative abundance of the five genera and related ASVs responding to the investigated early life determinants tested (antibiotics use during age 1–2 years (AB), exclusively breastfeeding (eBF), smoking during pregnancy, C-section (Csec), gender (female, male) and socioeconomic status (SES; high, ml)) (n = 166). Significant differences were calculated using multivariate ANOVA based on a linear mixed model (p value correction by Bonferroni method) and are indicated by asterisks (*p* < 0.05).

As expected the predominant bacterial phyla in all samples were *Firmicutes* (57.4%), *Bacteroidetes* (30.1%), *Verrucomicrobia* (4.6%), *Actinobacteria* (4.5%) and *Proteobacteria* (3.1%). Out of the total 225 bacterial genera detected, 23 genera showed relative abundance levels >1%. Five of these 23 genera were significantly influenced by early life determinants, mainly by antibiotics (AB) use and feeding practice (Fig. 2, S5 data). Interestingly, only genera belonging to *Firmicutes* and *Actinobacteria* were affected while the other phyla showed no response. This might explain why beta diversity was not significantly impacted by the early life determinants under investigation.

Members of *Bifidobacterium*, the predominant genus of *Actinobacteria*, were significantly enriched in infants treated with AB (4.5% of all reads) compared to non-treated infants (3.1%) (Fig. 2, S5 data). In total, 40 ASVs annotated to *Bifidobacterium* were detected, with three ASVs comprising 88.6% of all *Bifidobacterium*-related reads (S4 data). While ASV10 did not respond to any of the early life determinants, ASV20 was higher in children treated with AB compared to untreated infants. Furthermore, ASV20 was lower in eBF children compared to children not exclusively breastfed (Fig. 2). This inverse association with eBF was also visible at genus level. Reference sequences for all abundant ASVs are given in S6 data.

*Faecalibacterium*, the most abundant genus of *Firmicutes*, was represented by 133 ASVs, with three predominant ASVs accounting for 77.8% of all reads annotated to this genus (S4 data). While ASV1 was negatively affected by smoking during pregnancy and ASV5 was significantly higher in infants with high maternal SES, ASV4 responded to most of the early life determinants (Fig. 2, S5 data). ASV4 was positively affected by AB use (2.7% vs 1.9% of total reads) and C-section (3.2% vs. 2.1%) and significantly lower in eBF children (1.9%) compared to infants not eBF (3.0%). Additionally, it occurred in higher relative abundance in females (2.7%) compared to males (2.0%). Although not significant, comparable trends (positive effect of AB, C-section and high SES but negative effect of smoking) could be also observed for *Faecalibacterium* on genus level.

Members of *Blautia*, accounting for 5.1% of *Firmicutes*, were negatively affected by eBF (2.7% vs. 3.5% of total reads) and high SES (2.7% vs.3.9%) (Fig. 2). In total, 56 ASVs annotated to *Blautia* were observed, with three of them comprising 78.3% of all *Blautia*-related reads (S4 data). Whereas ASV31 and 56 remained unaffected by early life determinants, ASV26 was significantly lower in high SES infants and maternal non-smoking children and showed a negative trend towards eBF (Fig. 2, S5 data).

*Anaerostipes*, belonging to the family *Lachnospiraceae* like *Blautia*, showed a comparable negative trend in relative abundance towards eBF (1.7% vs.2.3%), too (Fig. 2, S5 data). Furthermore, the relative abundance of *Anaerostipes* spp. was significantly higher in infants treated with AB (2.3%) compared to untreated children (1.4%). In total, 33 ASVs of *Anaerostipes* were detected, with two predominating ASVs (78.2% of *Anaerostipes-*related reads) (S4 data). While ASV27 showed similar response towards AB (positive) and eBF (negative) as the total genus, ASV81 remained unaffected by early life determinants.

Members of *Christensenellaceae*_R7_group, accounting for 4.1% of *Firmicutes*, were represented by 122 ASVs, with two ASVs comprising 62.0% of *Christensenellaceae*_R7_group-related reads (S4 data). Whereas ASV40 did not respond to lifestyle factors, ASV24 was positively affected by eBF (1.2% vs. 0.6% of total reads) and high SES (1.9% vs. 0.5%). Similar responses towards eBF and SES were observed at genus level (Fig. 2, S5 data).

## Discussion

Although numerous studies investigated the impact of different lifestyle factors on the gut microbiome in early infancy^7–9,12^, knowledge on sustainable effects up to six years is still missing. This study focused on long-term effects of selected early life determinants (maternal smoking during pregnancy, mode of delivery, breastfeeding, antibiotics treatment during early infancy, gender and socioeconomic status) on the bacterial community structure of the gut microbiome of six-year-old children during the LISA birth cohort follow-up. The described overall composition of the gut microbiome at phylum level was comparable to the composition reported in a study where the gut microbiome of European children in the age of one to six years was analyzed^13^. The proportions for the most abundant phyla, *Firmicutes* and *Bacteroidetes*, were similar to the results in the present study.

Our results showed no influence of early life determinants on the overall composition of the gut microbiome (alpha and beta diversity). This is contrary to other studies who reported that especially feeding practices significantly affected microbial gut diversity of infants^14–16^. However, the lack of long-term effects on the overall gut microbiome is in line with a previous study describing that after 31 months a stable phase in children’s gut microbial progression is reached^17^. Taken together, this indicates that the age-related stabilization of the intestinal microbiome makes its responses to previous life exposures less visible, as they are superimposed by other factors with increasing age. In this respect it is remarkable that members of *Bacteriodetes*, whose abundance was previously reported as being affected by feeding practice and birth mode^16,17^, did not respond to any of the investigated factors and obviously contributed strongly to the observed resilience of the gut microbiome in our study.

Although alpha and beta diversity of the gut microbiome was not affected by the investigated factors, single genera and ASVs were significantly influenced, mainly by feeding practice and use of antibiotics. This is not surprising as breast milk with its pro- and prebiotic functions is known as one of the key factors determining the community composition of the infant gut microbiome^18^. Human milk oligosaccharides (HMOs) are only partly digested in the small intestine and thus mostly reach the colon where fermentation to short-chain fatty acids (SCFA) is catalyzed^19,20^. SCFA strongly trigger the interaction of bacteria with the gut epithelium^4^. *Bifidobacterium* spp. in particular, which are considered as important beneficial bacteria of the human gut, are known to trigger HMO fermentation and thus can be assumed to be positively associated with breastfeeding of infants as reported previously^21,22^. Interestingly, members of *Bifidobacterium* were negatively affected by eBF in our study on both genus and ASV level. Our results mirror the ongoing debate about the presence/predominance of *Bifidobacterium* in exclusively breastfed compared to non-exclusively breastfed or formula-fed infants: while numerous studies have reported a lower abundance of *Bifidobacterium* in formula-fed compared to breastfed infants^21,22^, several studies have reported *Bifidobacterium* being dominant in both feeding groups^23,24^. This controversy might be explained by age differences, but also by other lifestyle factors that contribute to the variation of the intestinal microbiome and become increasingly important over time, such as childhood infections, diet and living conditions^25^. On the other hand, species-specific adaptation to HMOs could occur within this genus: Praveen *et al*.^15^ observed that *B. breve* and *B. bifidum* was mostly detected in breastfed infants, whereas *B. longum* was detected in formula-fed infants. This confirms our result of significant lower relative abundance of ASV32 (annotated to *B. longum* via Basic Local Alignment Search Tool (BLAST) with query coverage 100% and identity 99.76%) in eBF children. Besides feeding practice, antibiotic treatment between the age of one to two years significantly influenced the relative abundance of *Bifidobacterium* on genus and ASV level in the present study. Surprisingly, AB use resulted in higher abundance of the genus *Bifidobacterium* and *Bifidobacterium*-related ASV20, which is contrary to previous reports in preschool children and adults suggesting that *Bifidobacterium* spp. are highly vulnerable to antibiotics^26,27^. However, Vatanen *et al*.^18^ observed in infants that antibiotic susceptibility of *Bifidobacterium* is species-specific: While for example the relative abundance of *B. bifidum, B. adolescentis* and *B. dentium* decreased after AB treatment, *B. breve* and *B. longum* remained unaffected. This suggests that certain *Bifidobacterium* species are more vulnerable to out-competition by other microbes after AB depletion than others. This is most likely due to dose and/or species-specific resistance towards certain AB. For example, some strains of *B. pseudocatnulatum* (top BLAST hit for ASV20 with query coverage 100% and identity 99.77%) are known to be resistant to tetracycline since they possess *tet* genes which protect ribosomes from the AB effect^28^. This implies that different types of antibiotics could act differently on the gut microbiome composition. Since no detailed information on type and dosage of antibiotics is available in our cohort, the elucidation of the mode of action for the persistence of selected species of the genus *Bifidobacterium* is not possible here.

Similar to *Bifidobacterium*, also the relative abundance of *Anaerostipes* was changed in association with antibiotics both on genus and ASV level. This is contradictory to the result of a Finnish cohort study, where *Anaerostipes* was reduced in preschool children exposed to macrolide AB within the last 6 months^26^. However, the effect of AB on relative abundance of *Aeaerostipes* might vary, on the one hand, due to the different time point of exposure: while the children from the Finnish cohort study were exposed to antibiotics prior to six months, the children from the current study were exposed four to five years ago and the information on antibiotic use in the months before stool sampling has not been collected in the present study. This long time span between AB treatment and sampling might have resulted in a higher influence of confounding factors like a more recent use of antibiotics, diet, childhood infections and other living conditions, which could have highly affected the present results. On the other hand, AB type and dosage might have been different between the studies.

Of note, members of *Faecalibacterium* responded to all investigated early life determinants in the present study. The genus *Faecalibacterium* belongs to the family of *Ruminococcaceae* and was the predominant genus of *Firmicutes*, comprising 9.1% of all reads. The sole species, *F. prausnitzii*, is considered as key marker for a healthy gut and has the ability to produce the SCFA butyrate by consuming acetate^29^. Produced butyrate is an energy source for the colonic epithelium and suppresses inflammation through G protein coupled receptors (GPCRs)^30^. As expected, non-smoking, vaginal birth and high SES, which are considered as beneficial, increased the relative abundance of *Faecalibacterium* on both genus and ASV level. Similar to *Bifidobacterium*, *Faecalibacterium* was associated negatively with exclusive breastfeeding but positively with antibiotics use. The first finding is in line with Thompson *et al*.^16^, who observed a higher relative abundance of *Faecalibacterium* in non-exclusively breastfed infants, too. Moreover, results of a Danish cohort study showed that colonization of *F. prausnitzii* in the gut microbiota of three-year-old children was not significantly influenced by breastfeeding^22^. The inverse association of antibiotic treatment and relative abundance of *Faecalibacterium* could be due to tolerance against certain antibiotics. According to the study of Foditsch *et al*.^31^, most isolated strains of *F. prausnitzii* from fecal samples of calves and piglets showed antibiotic resistance against ciprofloxacin and sulfamethoxazole-trimethoprim, and approximately 50% of isolated strains were resistant to tetracycline, amikacin, cefepime and cefoxitin. Although results from animal studies must not be representative for humans, a potential AB resistance would increase the competitiveness of *Faecalibacterium* towards other gut microbes in AB-treated infants and thus explain the increase in its relative abundance.

The strength of this study is the prospectively collected information on the early life determinants within the longitudinal LISA birth cohort study. Therefore, recall bias is assumed to be lower than in a retrospective cohort study^32^. In addition, the population is homogenous regarding age, region and ethnicity, which is expected to minimize the inter-individual variation of the gut microbiome. The absence of fecal samples collected in infancy could be considered as a limitation of this study since the comparison of the gut microbiome composition in infancy and childhood would allow studying the effects of early life determinants on the dynamics of the gut microbiome development during colonization and stabilization over time. Moreover, a comprehensive assessment of diet has not been conducted during the 6-year follow-up and also detailed information on the history of antibiotic use is missing. Other potential residual confounding factors could also affect the results of this study, since this observational study only account for a limited number of early life determinants. However, socio-economic status was included to account at least partially for some lifestyle factors related to it. Furthermore, some of the confounding factors investigated are assumed to be correlated like smoking, low SES and reduced breast feeding. Thus, it is not in all cases possible to clearly link the data to one of the early life determinants investigated in this study. However, the analysis of the gut microbiome composition after its stabilization indicates the persistence of early life determinant effects up to six years, although the effects were not as pronounced as reported for early infancy by other studies. The effects are probably overridden by other lifestyle and environmental factors, which become more important with increasing age.

The mechanisms by which early life factors could have a long-term effect on the gut microbiome composition are still unknown. One possible explanation could be that microorganisms colonize specific niches of the gut in early childhood. If those microbes are absent because of external stimuli, e.g. AB treatment, those niches would be colonized by other microorganisms. Once established, microbes form complex networks, leading to an altered but nonetheless stable gut microbiome, which is only modifiable by major lifestyle changes.

However, this observational study allows only to report correlations and to indicate specific organisms which might be relevant for later health and thereby contribute to an early detection of populations at risk. The identification of underlying mechanisms is beyond the scope of the present study and requires well designed and controlled animal studies, or a metagenomic approach considering the host’s phenotype.

## Conclusion

Our findings indicate that early life determinants could have a long-term sustainable effect on the gut microflora of six-year-old children, but associations are weaker than reported for infants. No significant effects of early life determinants on the overall composition of the gut microbiome (alpha and beta diversity) were observed. Surprisingly, gut microbes that are often considered as beneficial like *Bifidobacterium* and *Faecalibacterium* were unaltered or even positively affected by less breastfeeding or antibiotics use. This indicates that microbial responses are far more on the level of species or even ecotypes while the level of genera and families is simply too diverse to predict common response pattern towards early life determinants. Therefore, further research is needed to explore the association between early life determinants and lasting changes of the gut microbiome composition including levels beyond genera and families. Second, a detailed understanding how changes of the host’s microbiome affect the education of immune responses in early life is required. Finally, a causal relationship with health outcomes such as allergies or asthma needs to be proven. This information can then be utilized to develop preventative or other health promotion strategies which beneficially shape the gut microbiota composition, such as through pro- or prebiotics to increase the abundance of certain species, thereby mitigating the negative effect of lifestyle factors.

## Methods

### Study population and sampling

The present study is based on the LISA (Influence of Life-style factors on the development of the Immune System and Allergies in East and West Germany) birth cohort. Subjects of the LISA birth cohort study were recruited from 1997 to 1999 in four regions of Germany (n = 3,097). Recruitment and exclusion criteria have been described in previous publications^33,34^. The LISA study has been conducted in line with the national research regulations in place at the time of data collection and was approved by local ethics committees (Bavarian General Medical Council, University of Leipzig, and the Medical Council of North Rhine-Westphalia). Written informed consent was obtained from all parents or legal guardians.

Stool samples from 166 six-year-old children from the Munich study center were collected during the follow-up in 2003 to 2005. Only participants who attended the physical examination in the study center and completed all questionnaires up to the 6-year follow-up were included. Stool samples were collected at home with sampling kits provided to the families during the physical examination. The samples were delivered immediately to the study center and frozen at −20 °C. After completion of the sampling period, the samples were transferred and stored at −80 °C until analysis. Information on early life determinants and potential confounding factors were collected from questionnaires administered to the parents. In total, six factors were analyzed in this study. The four early life determinants included in this study were maternal smoking during pregnancy, mode of delivery, exclusive breastfeeding (eBF) for at least four months and antibiotics (AB) treatment in early life (during one to two years of age), while two potential confounding factors were gender and socioeconomic status (SES; using maternal level of education as a proxy). A detailed description of determinant assessment is given in the Supplement (S1 data).

### 16S rRNA gene amplicon sequencing

Approximately 125 mg fecal sample was used for DNA extraction using the QIAMP® Power Fecal DNA kit according to the manufacturer’s instructions (Qiagen, Hilden, Germany). Potential DNA contaminants in the extraction kit were analyzed by including negative extraction controls using water instead of fecal material for extraction. The quantity of the extracted DNA was measured using the Quant-iT Picogreen kit (Thermofisher, Waltham, USA). The V3–V4 region of the 16S rRNA gene was used as target for amplification. The PCR was performed using the NEBNext® high fidelity polymerase (New England Biolabs, Ipswich, USA) and primer pair S-D-Bact-0341-b-S-17, 5′-CCTACGGGNGGCWGCAG-3′, and S-D-Bact-0785-a-A-21, 5′-GACTACHVGGGTATCTAATCC-3^35^, in a total volume of 25 µl (10 ng DNA template, 12.5 µl polymerase, 0.5 pmol of each primer pair). PCR conditions were 30 s at 98 °C; 25 cycles of 10 s at 98 °C, 30 s at 55 °C, 30 s at 72 °C; 5 min 72 °C. PCR products were qualified using gel electrophoresis and purified using Agencourt AMPure® XP beads (Beckman Coulter, Brea, CA, US). The DNA concentration was measured as described above and the quality was checked using a Fragment Analyzer™ (Advanced Analytical Technologies, Inc., Ankeny, USA). The subsequent indexing PCR was performed using the Nextera XT Index Kit v2 (Illumina, Inc. San Diego, CA, US) and the NEBNext® high fidelity polymerase (New England Biolabs, Ipswich, USA) in a total volume of 25 µl (10 ng DNA template, 12.5 µl polymerase, 2.5 µl of each index) and the following PCR conditions: 30 s at 98 °C; 8 cycles of 10 s at 98 °C, 30 s at 55 °C, 30 s at 72 °C; 5 min 72 °C. Indexing PCR products were purified and quantified as described above and pooled in an equimolar ratio of 4 nM. Sequencing was performed using an Illumina Miseq platform (Illumina Inc., San Diego, USA) with Reagent Kit v3 (600 cycles). The nucleotide sequence data obtained in this study can be provided to interested researchers by the corresponding author upon reasonable request, provided the release is consistent with the consent given by the LISA study participants. Ethical approval might be obtained for the release and a data transfer agreement from the legal department of the Helmholtz Zentrum München must be accepted.

### Sequencing processing

Sequences were analyzed using the QIIME 2 software package release 2017.11^36^ with default parameters. FASTQ files were trimmed and merged with a minimum read length of 50 and minimum Phred score of 15 using AdapterRemoval^37^. Quality control was done using the QIIME 2 plugin DADA2^38^ by removing 10 bp n-terminally and truncating the reads at position 280 (forward) and 220 (reverse), respectively, resulting in unique amplicon sequence variants (ASVs). ASVs are biological sequences discriminated from errors, allowing the detection of single-nucleotide differences over the sequenced gene. The ASV method has demonstrated a better specificity and sensitivity than OTU based methods, which cluster sequencing reads according a fixed dissimilarity threshold^39^. For taxonomic analysis, Naive Bayes classifier was pre-trained on the SILVA_132_QIIME release 99% using the primer pair S-D-Bact-0341-b-S-17/S-D-Bact-0785-a-A-21. To exclude potential contamination, ASVs occurring in extraction control and PCR no template control samples (15 ASVs in total) were removed from dataset.

### Statistical analysis

All statistical analyses were performed using R version 3.5.0 (https://www.R-project.org). The analyzed subsample set (n = 166) was compared to the total Munich cohort (n = 1298) to confirm the representativeness of the data set using a Fisher’s exact test. Species evenness and within species diversity were measured using Pielou evenness^40^, Shannon diversity and Simpson’s diversity index^41^. Species richness was evaluated based on the number of observed ASVs^38^. Statistical analysis of the gut microbiome composition was done using either the nonparametric Kruskal-Wallis test for multiple comparisons and *p*-value correction by the Benjamini-Hochberg method or, when homogeneity of variance was given, permutational multivariate analysis of variance (ANOVA, Adonis function) based on Yue Clayton dissimilarity, Euclidean distances of Hellinger transformed data, Jaccard distance, Bray-Curtis distance and both weighted and unweighted Unifrac distance. Additionally, a linear mixed model was calculated to test for significant effects among single taxa (including p value correction for multiple testing by Bonferroni method). The following R packages were used for analyses and visualization: ade4, agricolae, cluster, ggplot2, gridExtra, lme4, mass, plyr, qiime2R, reshape2, tidyverse and vegan.

## Supplementary information

Supplementary Information

## Data Availability

Due to data protection reasons, the datasets generated and/or analyzed during the current study cannot be made publicly available. The datasets are available to interested researchers from the corresponding author on reasonable request (e.g. reproducibility), provided the release is consistent with the consent given by the LISA study participants. Ethical approval might be obtained for the release and a data transfer agreement from the legal department of the Helmholtz Zentrum München must be accepted.
