# Supplementary material for: Early life determinants induce sustainable changes in the gut microbiome of six-year-old children
Source: Sci Rep. 2019 Sep 3;9:12675. doi: 10.1038/s41598-019-49160-7 (PMC6722248; doi:10.1038/s41598-019-49160-7)
Supplement: Supplementary file 1 — Supplementary Information [file 41598_2019_49160_MOESM1_ESM.pdf]

Early life determinants induce sustainable changes in the gut microbiome of six-year-old children

Authors: Silvia Gschwendtner[1], Hyena Kang[1], Elisabeth Thiering[2,3], Susanne Kublik[1], Bärbel Fösel[1], Holger Schulz[2], Susanne Krauss-Etschmann[4,5], Joachim Heinrich[2,6], Anne Schöler[1,7,8,9], Michael Schloter[1,10]*, Marie Standl[2]*

* These authors jointly directed this work.

Affiliation:

1. Research Unit for Comparative Microbiome Analysis, Helmholtz Zentrum München, German Research Center for Environmental Health, Ingolstädter Landstr. 1, 85764 Neuherberg, Germany

2. Institute of Epidemiology, Helmholtz Zentrum München, German Research Center for Environmental Health, Ingolstädter Landstr. 1, 85764 Neuherberg, Germany

3. Division of Metabolic and Nutritional Medicine, Dr. von Hauner Children's Hospital, University of Munich Medical Center, Lindwurmstraße 4, 80337 Munich, Germany

4. Institute for Experimental Medicine, Christian-Albrechts-Universität zu Kiel, Niemannsweg 11, 24105, Kiel, Germany

5. Division of Experimental Asthma Research, Research Center Borstel, Leibniz Lung Center, Member of the German Center for Lung Research (DZL), Parkallee 1-40, 23845 Borstel, Germany

6. Institute and Clinic for Occupational, Social and Environmental Medicine, University Hospital, LMU Munich, Ziemssenstr. 1, 80336 Munich, Germany

7. Institute for Neuropathology, Charité, Charitéplatz 1, 10117 Berlin, Germany

8. German Cancer Consortium (DKTK), Germany

9. German Cancer Research Center (DKFZ), Im Neuenheimer Feld 280, 69120 Heidelberg, Germany

10. Technical University of Munich, Zentral Insititut for Food and Health (ZIEL), Weihenstephaner Berg 1, 85354 Freising, Germany

Corresponding author

Dr. Marie Standl

Helmholtz Zentrum München GmbH

Institute of Epidemiology

Ingolstädter Landstr. 1

85764 Neuherberg

Germany

Tel.: +498931872952

Fax: +498931873380

Email: marie.standl@helmholtz-muenchen.de

Supplementary data S1. Definition of early life determinants

Maternal smoking: Information on the number of cigarettes smoked by the mother on average during each trimester was collected at birth. Smoking during any trimester of pregnancy was categorized as “yes” and the absence of smoking in the entire pregnancy was allocated to “no.”

C-section: The information on the mode of delivery was collected at birth. C-section was allocated as “yes” and natural birth and vaginal operation were allocated as “no.”

Breastfeeding: At six months of age, the parents were asked for the infant feeding mode for each of the first six months of life. Possibly categories were “exclusive breastfeeding” (eBF), “exclusive formula feeding” or “mixed breast and formula feeding”. Feeding mode was allocated to two groups. eBF for at least four months were categorized as ”yes” and otherwise categorized as ”no” (infant formula or mixed feeding).

Antibiotics (AB): Information on antibiotics use in the past 6 months was collected during the 18 months and 24 months follow-ups. The antibiotic usage at any time point was defined as “yes” and non-usage of antibiotics or unknown cases were defined as “no.”

Potential confounding factors

Gender: information on the gendergender of the participants was obtained at birth.

Socioeconomic status (SES): SES was categorized based on the maternal education level. The information on maternal education was collected from participants’ mothers at birth by self-administered questionnaires. Participants were categorized as “low” (less than 10th grade), “medium” (10th grade) or “high” (more than 10th grade) according to the Germany education system. SES was allocated to two groups: high and medium/low (ml).

Supplementary data S2. Comparison between the analyzed sample and remaining population from Munich study center

	Munich study center only without analysis sample	Analysis sample	*p*-values[a]
	% (n)		
**N**	89% (1298)	11% (166)	-
**Gender - females**	48% (620)	44% (73)	0.37
**Exclusively breastfed for >= 4 months**	63% (727)	71% (111)	0.06
**Caesarean section**	19% (241)	16% (27)	0.52
**Antibiotic use (between age 1-2 yrs)**	44% (504)	51% (84)	0.09
**Maternal smoking during pregnancy**	16% (196)	11% (18)	0.19
**Socioeconomic status - high**	64% (816)	66% (109)	0.61

[a] *p*-values were obtained from Fisher's exact test.

Supplementary data S3. Subsampling depth.

Rarefaction curve displays the number of observed ASVs by the number of sequencing depth of all samples. Blue lines indicate samples (n= 166).

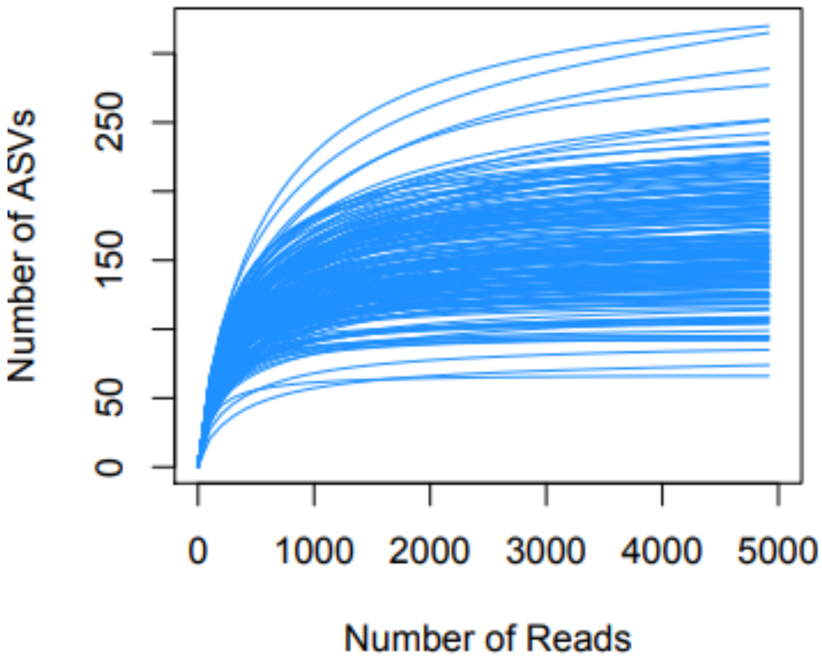

Supplementary data S4. ASV composition of the five genera responding to the investigated early life determinants tested.

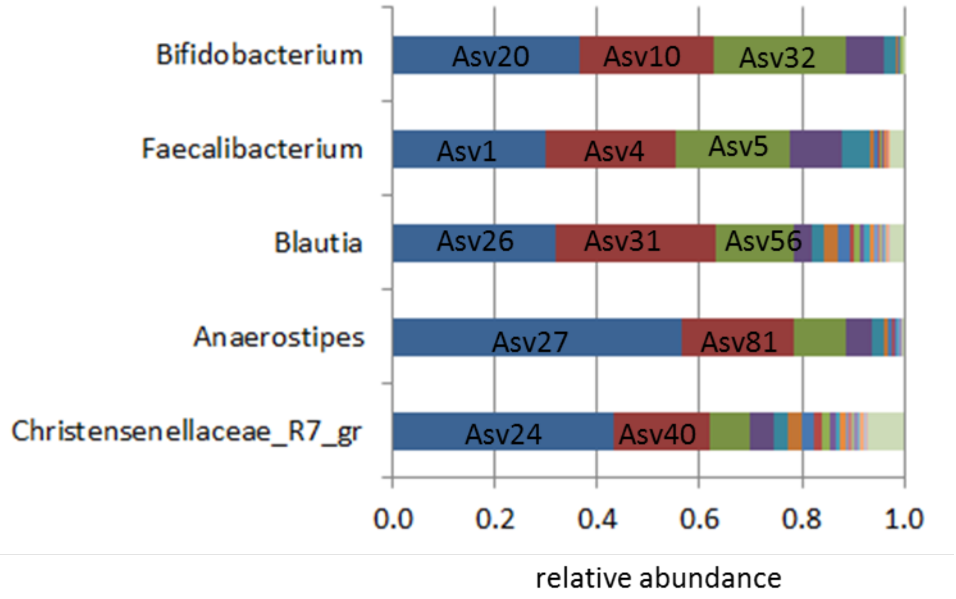

Supplementary data S5. p values showing significant response of taxa and ASVs to early life determinants. Investigated factors are: antibiotics use (AB), exclusive breastfeeding (eBF), smoking during pregnancy, C-section (Csec), gender and socioeconomic status (SES). Significant differences were calculated using linear models and p value correction by Bonferroni and are indicated by bold letters ($p < 0.05$).

	AB	eBF	Smoking	Csec	Gender	SES
Bifidobacterium	**0.036**	0.061	0.896	0.417	0.446	0.124
Asv20_*Bifidobacterium*	**0.032**	0.783	0.930	0.868	0.595	0.870
Asv32_*Bifidobacterium*	0.142	**0.003**	0.847	0.266	**0.004**	0.627
Faecalibacterium	0.163	0.443	0.156	0.145	**0.005**	0.944
Asv1_*Faecalibacterium*	0.444	0.378	**0.013**	0.997	0.061	0.143
Asv4_*Faecalibacterium*	**0.041**	**0.001**	0.260	**0.003**	**0.006**	0.099
Asv5_*Faecalibacterium*	0.500	0.761	0.449	0.813	0.167	**0.008**
Blautia	0.946	**0.021**	0.347	0.956	0.917	**0.019**
Asv26_*Blautia*	0.261	0.248	**0.006**	0.473	0.748	**0.017**
Anaerostipes	**0.011**	0.059	0.645	0.523	0.111	0.320
Asv27_*Anaerostipes*	**0.008**	**0.012**	0.789	0.453	0.120	0.154
Christensenellaceae R-7 group	0.452	**0.049**	0.666	0.455	0.134	0.200
Asv24_Christensenellaceae R-7 group	0.583	**0.022**	0.091	0.490	0.649	**0.030**

Supplementary data S6. Reference sequences of abundant and/or significant ASVs

Asv1
GCTGCAGTGGGGAATATTGCACAATGGGGGAAACCCTGATGCAGCGACGCCGCGTGGAGGAAGAAGGTCTTCGGATTGT<br>AAACTCCTGTTGTTGAGGAAGATAATGACGGTACTCAACAAGGAAGTGACGGCTAACTACGTGCCAGCAGCCGCGGTAAA<br>ACGTAGGTCACAAGCGTTGTCCGGAATTACTGGGTGTAAAGGGAGCGCAGGCGGGAGAACAAGTTGGAAGTGAAATCCAT<br>GGGCTCAACCCATGAACTGCTTTCAAAACTGTTTTTCTTGAGTAGTGCAGAGGTAGGCGGAATTCCCGGTGTAGCGGTGG<br>AATGCGTAGATATCGGGAGGAACACCAGTGGCGAAGGCGGCCTACTGGGCACCAACTGACGCTGAGGCTCGAAAGTGTG<br>GGTAGCAAACAGGATTAGATAC
Asv4
GCTGCAGTGGGGAATATTGCACAATGGGGGAAACCCTGATGCAGCGACGCCGCGTGGAGGAAGAAGGTCTTCGGATTGT<br>AAACTCCTGTTGTTGGGGAAGATAATGACGGTACCCAACAAGGAAGTGACGGCTAACTACGTGCCAGCAGCCGCGGTAAA<br>ACGTAGGTCACAAGCGTTGTCCGGAATTACTGGGTGTAAAGGGAGCGCAGGCGGGAAGACAAGTTGGAAGTGAAATCTAT<br>GGGCTCAACCCATAAACTGCTTTCAAAACTGTTTTTCTTGAGTAGTGCAGAGGTAGGCGGAATTCCCGGTGTAGCGGTGGA<br>ATGCGTAGATATCGGGAGGAACACCAGTGGCGAAGGCGGCCTACTGGGCACCAACTGACGCTGAGGCTCGAAAGTGTGG<br>GTAGCAAACAGGATTAGATAC
Asv5
GCTGCAGTGGGGAATATTGCACAATGGGGGAAACCCTGATGCAGCGACGCCGCGTGGAGGAAGAAGGTCTTCGGATTGT<br>AAACTCCTGTTGTTGAGGAAGATAATGACGGTACTCAACAAGGAAGTGACGGCTAACTACGTGCCAGCAGCCGCGGTAAA<br>ACGTAGGTCACAAGCGTTGTCCGGAATTACTGGGTGTAAAGGGAGCGCAGGCGGGAAGACAAGTTGGAAGTGAAATCTAT<br>GGGCTCAACCCATAAACTGCTTTCAAAACTGTTTTTCTTGAGTAGTGCAGAGGTAGGCGGAATTCCCGGTGTAGCGGTGGA<br>ATGCGTAGATATCGGGAGGAACACCAGTGGCGAAGGCGGCCTACTGGGCACCAACTGACGCTGAGGCTCGAAAGTGTGG<br>GTAGCAAACAGGATTAGATAC
Asv10
GCTGCAGTGGGGAATATTGCACAATGGGCGCAAGCCTGATGCAGCGACGCCGCGTGCGGGATGACGGCCTTCGGGTTGT<br>AAACCGCTTTTGACTGGGAGCAAGCCCTTCGGGGTGAGTGTACCTTTCGAATAAGCACCGGCTAACTACGTGCCAGCAGC<br>CGCGGTAATACGTAGGGTGCAAGCGTTATCCGGAATTATTGGGCGTAAAGGGCTCGTAGGCGGTTCGTCGCGTCCGGTG<br>TGAAAGTCCATCGCTTAACGGTGGATCCGCGCCGGGTACGGGCGGGCTTGAGTGCGGTAGGGGAGACTGGAATTCCCGG<br>TGTAACGGTGGAATGTGTAGATATCGGGAAGAACACCAATGGCGAAGGCAGGTCTCTGGGCCGTCACTGACGCTGAGGA<br>GCGAAAGCGTGGGGAGCGAACAGGATTAGATAC
Asv20
GCTGCAGTGGGGAATATTGCACAATGGGCGCAAGCCTGATGCAGCGACGCCGCGTGCGGGATGACGGCCTTCGGGTTGT<br>AAACCGCTTTTGATCGGGAGCAAGCCTTCGGGTGAGTGTACCTTTCGAATAAGCACCGGCTAACTACGTGCCAGCAGCCG<br>CGGTAATACGTAGGGTGCAAGCGTTATCCGGAATTATTGGGCGTAAAGGGCTCGTAGGCGGTTCGTCGCGTCCGGTGTGA<br>AAGTCCATCGCTTAACGGTGGATCTGCGCCGGGTACGGGCGGGCTGGAGTGCGGTAGGGGAGACTGGAATTCCCGGTGT<br>AACGGTGGAATGTGTAGATATCGGGAAGAACACCAATGGCGAAGGCAGGTCTCTGGGCCGTTACTGACGCTGAGGAGCG<br>AAAGCGTGGGGAGCGAACAGGATTAGATAC
Asv32
GCTGCAGTGGGGAATATTGCACAATGGGCGCAAGCCTGATGCAGCGACGCCGCGTGAGGGATGGAGGCCTTCGGGTTGT<br>AAACCTCTTTTATCGGGGAGCAAGCGAGAGTGAGTTTACCCGTTGAATAAGCACCGGCTAACTACGTGCCAGCAGCCGCG<br>GTAATACGTAGGGTGCAAGCGTTATCCGGAATTATTGGGCGTAAAGGGCTCGTAGGCGGTTCGTCGCGTCCGGTGTGAAA<br>GTCCATCGCTTAACGGTGGATCCGCGCCGGGTACGGGCGGGCTTGAGTGCGGTAGGGGAGACTGGAATTCCCGGTGTAA<br>CGGTGGAATGTGTAGATATCGGGAAGAACACCAATGGCGAAGGCAGGTCTCTGGGCCGTTACTGACGCTGAGGAGCGAA<br>AGCGTGGGGAGCGAACAGGATTAGATAC
Asv26
GCTGCAGTGGGGAATATTGCACAATGGGGGAAACCCTGATGCAGCGACGCCGCGTGAAGGAAGAAGTATCTCGGTATGT<br>AAACTTCTATCAGCAGGGAAGATAATGACGGTACCTGACTAAGAAGCCCCGGCTAACTACGTGCCAGCAGCCGCGGTAAT<br>ACGTAGGGGGCAAGCGTTATCCGGATTTACTGGGTGTAAAGGGAGCGTAGACGGCGCAGCAAGTCTGATGTGAAAGGCA<br>GGGGCTTAACCCCTGGACTGCATTGGAAACTGCTGTGCTTGAGTGCCGGAGGGGTAAGCGGAATTCCTAGTGTAGCGGT<br>GAAATGCGTAGATATTAGGAGGAACACCAGTGGCGAAGGCGGCTTACTGGACGGTAACTGACGTTGAGGCTCGAAAGCGT<br>GGGGAGCAAACAGGATTAGATAC
Asv31
GCTGCAGTGGGGAATATTGCACAATGGGGGAAACCCTGATGCAGCGACGCCGCGTGAAGGAAGAAGTATCTCGGTATGT<br>AAACTTCTATCAGCAGGGAAGAAAATGACGGTACCTGACTAAGAAGCCCCGGCTAACTACGTGCCAGCAGCCGCGGTAAT<br>ACGTAGGGGGCAAGCGTTATCCGGATTTACTGGGTGTAAAGGGAGCGTAGACGGATGGACAAGTCTGATGTGAAAGGCT<br>GGGGCTCAACCCCGGGACTGCATTGGAAACTGCCCGTCTTGAGTGCCGGAGAGGTAAGCGGAATTCCTAGTGTAGCGGT<br>GAAATGCGTAGATATTAGGAGGAACACCAGTGGCGAAGGCGGCTTACTGGACGGTAACTGACGTTGAGGCTCGAAAGCGT<br>GGGGAGCAAACAGGATTAGATAC
Asv56
GCTGCAGTGGGGAATATTGCACAATGGGGGAAACCCTGATGCAGCGACGCCGCGTGAAGGAAGAAGTATCTCGGTATGT<br>AAACTTCTATCAGCAGGGAAGATAGTGACGGTACCTGACTAAGAAGCCCCGGCTAACTACGTGCCAGCAGCCGCGGTAAT<br>ACGTAGGGGGCAAGCGTTATCCGGATTTACTGGGTGTAAAGGGAGCGTAGACGGACTGGCAAGTCTGATGTGAAAGGCG<br>GGGGCTCAACCCCTGGACTGCATTGGAAACTGTTAGTCTTGAGTGCCGGAGAGGTAAGCGGAATTCCTAGTGTAGCGGTG<br>AAATGCGTAGATATTAGGAGGAACACCAGTGGCGAAGGCGGCTTACTGGACGGTAACTGACGTTGAGGCTCGAAAGCGTG<br>GGAGCAAACAGGATTAGATAC

Asv27
GCTGCAGTGGGGAATATTGCACAATGGGGGAAACCCTGATGCAGCGACGCCGCGTGAGTGAAGAAGTATCTCGGTATGTA<br>AAGCTCTATCAGCAGGGAAGAAAATGACGGTACCTGACTAAGAAGCCCCGGCTAACTACGTGCCAGCAGCCGCGGTAATA<br>CGTAGGGGGCAAGCGTTATCCGGAATTACTGGGTGTAAAGGGTGCGTAGGTGGTATGGCAAGTCAGAAGTGAAAACCCA<br>GGGCTTAACTCTGGGACTGCTTTTGAAACTGTCAGACTGGAGTGCAGGAGAGGTAAGCGGAATTCCTAGTGTAGCGGTGA<br>AATGCGTAGATATTAGGAGGAACATCAGTGGCGAAGGCGGCTTACTGGACTGAAACTGACACTGAGGCACGAAAGCGTGG<br>GGAGCAAACAGGATTAGATAC
Asv81
GCTGCAGTGGGGAATATTGCACAATGGGGGAAACCCTGATGCAGCGACGCCGCGTGAGTGAAGAAGTATCTCGGTATGTA<br>AAGCTCTATCAGCAGGGAAGAAAATGACGGTACCTGACTAAGAAGCCCCGGCTAACTACGTGCCAGCAGCCGCGGTAATA<br>CGTAGGGGGCAAGCGTTATCCGGAATTACTGGGTGTAAAGGGTGCGTAGGTGGTATGGCAAGTCAGAAGTGAAAACCCA<br>GGGCTTAACTCTGGGACTGCTTTTGAAACTGTCAGACTAGAGTGCAGGAGAGGTAAGCGGAATTCCTAGTGTAGCGGTGA<br>AATGCGTAGATATTAGGAGGAACATCAGTGGCGAAGGCGGCTTACTGGACTGAAACTGACACTGAGGCACGAAAGCGTGG<br>GGAGCAAACAGGATTAGATAC
Asv24
GCTGCAGTGGGGAATATTGGGCAATGGAGGAAACTCTGACCCAGCAACGCCGCGTGGAGGAAGAAGGTTTTCGGATCGT<br>AAACTCCTGTCCTTGGAGACGAGTAGAAGACGGTATCCAAGGAGGAAGCCCCGGCTAACTACGTGCCAGCAGCCGCGGT<br>AATACGTAGGGGGCAAGCGTTGTCCGGAATAATTGGGCGTAAAGGGCGCGTAGGCGGCTCGGTAAGTCTGGAGTGAAAG<br>TCCTGCTTTTAAGGTGGGAATTGCTTTGGATACTGTCGGGCTTGAGTGCAGGAGAGGTTAGTGGAATTCCCAGTGTAGCG<br>GTGAAATGCGTAGAGATTGGGAGGAACACCAGTGGCGAAGGCGACTAACTGGACTGTAACTGACGCTGAGGCGCGAAAG<br>TGTGGGGAGCAAACAGGATTAGATAC
Asv40
GCTGCAGTGGGGAATATTGGGCAATGGGGGGAACCCTGACCCAGCAACGCCGCGTGGAGGAAGAAGGTTTTCGGATCGT<br>AAACTCCTGTCCTTGGAGACGAGTAGAAGACGGTATCCAAGGAGGAAGCCCCGGCTAACTACGTGCCAGCAGCCGCGGT<br>AATACGTAGGGGGCAAGCGTTGTCCGGAATAATTGGGCGTAAAGGGCGCGTAGGCGGCTCGGTAAGTCTGGAGTGAAAG<br>TCCTGCTTTTAAGGTGGGAATTGCTTTGGATACTGTCGGGCTTGAGTGCAGGAGAGGTTAGTGGAATTCCCAGTGTAGCG<br>GTGAAATGCGTAGAGATTGGGAGGAACACCAGTGGCGAAGGCGACTAACTGGACTGTAACTGACGCTGAGGCGCGAAAG<br>TGTGGGGAGCAAACAGGATTAGATAC
